# Enhanced ERbeta immunoexpression and apoptosis in the germ cells of cimetidine-treated rats

**DOI:** 10.1186/1477-7827-7-127

**Published:** 2009-11-18

**Authors:** Estela Sasso-Cerri

**Affiliations:** 1 Department of Morphology, Laboratory of Histology and Embryology, Dental School of São Paulo State University, Rua Humaitá, 1680, CEP: 14801-903, Araraquara (São Paulo), Brazil

## Abstract

**Background:**

Cimetidine, refereed as antiandrogenic drug, causes hormonal changes in male patients such as increased testosterone and FSH levels. In the rat testis, structural alterations in the seminiferous tubules have been related to germ cell loss and Sertoli cell death by apoptosis. Regarding the important role of Sertoli cells in the conversion of testosterone into estrogen, via aromatase, the immunoexpression of estrogen receptors-beta (ERbeta) was evaluated in the germ cells of untreated and treated rats with cimetidine. A relationship between ERbeta immunoreactivity and apoptosis was also investigated in the germ cells of damaged tubules.

**Methods:**

Immunohistochemistry for detection of ERbeta and TUNEL method were performed in testicular sections of adult male rats treated with 50 mg/Kg of cimetidine (CmG) or saline solution (CG) for 52 days.

**Results:**

In CG, a cytoplasmic immunoexpression for ERbeta was observed in spermatogonia, primary spermatocytes and spermatids. An evident ERbeta immunoreactivity was always observed in the flagellum and residual bodies of late spermatids. In CmG, the cytoplasm or cytoplasm and nuclei of germ cells of the damaged tubules by cimetidine showed enhanced ERbeta immunostaining. TUNEL-labeling was usually observed in the same germ cell types exhibiting enhanced ERbeta immunoreactivity.

**Conclusion:**

The presence of ERbeta immunolabeling in the flagellum and residual bodies of spermatids reinforces the role of estrogen in spermiogenesis. The overexpression of ERbeta in the germ cells of CmG could be related to a possible interference of cimetidine on tubular androgenization and/or on the intratubular aromatase due to Sertoli cell damage. The parallelism between ERbeta overexpression and apoptosis indicates a participation of ERbeta on germ cell death.

## Background

Additionally to testosterone, studies have demonstrated that estrogens play also a role in the local regulation of spermatogenesis [1-6]. Testosterone is converted into estrogens via cytochrome P450 - an aromatase enzyme [7]. In the testis, this enzyme is present in Sertoli and Leydig cells [1,8], and has also been detected in germ cells [1,3,4,8], suggesting that estrogen is locally produced from testosterone in the seminiferous epithelium. It has been demonstrated that the effect of estrogen action on the reproductive system is mediated by two estrogen receptors, ERα and ERβ [4,6,9-11]. In the testis, ERβ is significantly expressed in comparison to ERα, mainly in the germ cells [12]. This type of receptor has been detected in germ cells of humans [13-17], rodents [2,10,18-20], and other mammalian species [11,21], indicating that estrogens play a physiological role in the spermatogenic process via ERβ.

In young and adult rodents, ERβ has been detected immunohistochemically in gonocytes, spermatogonia [2,18], pachytene spermatocytes and spermatids [2,10,18,20]. The presence of ERβ in spermatocytes, round [2,10,18] and elongate spermatids [3] has indicated a role of estrogens on spermatid maturation. This role has been reinforced by the fact that deficiency of aromatase leads to reduction in the number of spermatids [22]. On the other hand, low doses of estrogen can potentially cause severe spermatogenic cellular dysfunction [23]. Estrogen induces up-regulation of Fas and FasL in adult rat testis, resulting in the germ cell apoptosis [24]. It has been demonstrated that either extrinsic (cell death receptors) or intrinsic (mitochondria) pathways are involved in the estrogen-induced germ cell apoptosis [23].

Cimetidine is an H_2_-receptor antagonist that inhibits acid secretion and is clinically used for the treatment of gastric and duodenal ulcers [25]. However, some adverse effects have been described in male patients: a) loss of libido and impotence; 2) increased levels of FSH and testosterone [26] and 3) gynaecomastia [27]. The effects of cimetidine in adult castrated male rats androgenized with testosterone revealed a significant decrease in ventral prostate and seminal vesicle weights [28]. Moreover, this drug competes for tritiated dihydrotestosterone-binding sites in mouse kidney preparations [29]. Thus, this drug has demonstrated to be an anti-androgenic agent, competing for androgen receptors [28-30].

In male rats, cimetidine has caused increased FSH levels [31], reduction in testicular weight [32,33] and structural alterations in the seminiferous tubules [31,33-36], including loss of germ cells by apoptosis [35]. The tubular alterations have suggested a possible antiandrogenic effect of cimetidine on the tubular androgenization [33,34]. Besides these effects, cimetidine induces peritubular myoid cell death [31,35] and structural alterations in the Sertoli cell-basement membrane interface leading to Sertoli cell apoptosis [36]. Considering the antiandrogenic effect of cimetidine and the important role of Sertoli cells in the conversion of testosterone into estrogen, via aromatase, the immunoexpression of estrogen receptors (ERβ) in the germ cells of untreated and treated rats with cimetidine was evaluated. A relationship between ERβ immunoreactivity and apoptosis was also investigated in the germ cells of damaged tubules.

## Methods

### Animals and treatment

Ten adult Holtzman male rats weighing 250-300 g were maintained at 25°C, standard lighting conditions (12-h light/dark cycle), fed laboratory rat chow and given water *ad libitum*. The animals were grouped in control (CG) and cimetidine (CmG) groups containing five animals each. The animals from CmG received daily intraperitoneal injections containing aqueous solution of 50 mg of cimetidine (Tagamet^®^, SmithKline Beecham, Brazil) per kg of body weight for 52 days, period of a complete spermatogenic cycle [37]. The animals from CG received saline solution by the same route. Principles of laboratory animal care and national laws on animal use were observed. The protocol of this study was authorized by Ethical Committee for Animal Research of the Dental School of São Paulo State University (UNESP-Araraquara).

### Light microscopy

The animals were anaesthetised and sacrificed with chloral hydrate and the testes were fixed in 4% formaldehyde (freshly prepared from paraformaldehyde) buffered at pH 7.2 with 0.1 M sodium phosphate for 48 hours at room temperature. Subsequently, the specimens were dehydrated in graded ethanol and embedded in paraffin for detection of ERβ by immunohistochemistry and cell death by TUNEL method. Pieces of uterus from female rats were fixed in the same fixative and embedded in paraffin to be used as positive control for the immunohistochemistry.

### Immunohistochemistry for ERβ

Testicular and uterine sections - used as positive control [38], were adhered to silanized slides, hydrated and maintained in citrate buffer (0.001 M; pH 6.0), for 20 min., in a microwave oven (90°C) for activation of antigen. The sections were immersed in 3% hydrogen peroxide (10 min.) for inactivation of endogenous peroxide. After washings in Tris-HCl 0.05 M; pH 7.5 buffer (TBS), the sections were incubated in primary antibodies (rabbit polyclonal IgG anti-rat estrogen receptor-β; Upstate Cell Signaling Solutions; Lake Placid, NY, USA), 1:200, diluted in TBS and 5% BSA, at 4°C. After 14 h, the sections were washed in TBS and incubated in solution containing biotinylated anti-rabbit antibodies (LSAB-plus kit; DaKO Corporation, USA) for 30 min. at room temperature, washed in TBS and incubated with streptavidin-peroxidase complex (LSAB-plus kit; DAKO) for 15 min at room temperature. After washings in TBS, the reaction was revealed with 0,06% 3.3'-diaminobenzidine tetrahydrochloride (DAB - Sigma-Aldrich Chemical Co., St. Louis, USA). The sections were counterstained with haematoxylin, dehydrated and mounted in resin. To be sure that the immunoreaction could not be resulted from unspecific staining, testicular sections from CG and CmG, used as negative controls, were performed following the same protocol, except that the incubation in the primary antibody was replaced by incubation in non-immune serum. The morphological analysis and capture of images were made by using light microscopes (Olympus, BX-50 and BX-51).

### Evaluation of the immunoreactivity

Four testicular sections per animal from CG and CmG were used. In each section, the ERβ-positive germ cells in six seminiferous tubules per section were analyzed according to the intensity of ERβ immunolabeling and scored as weak (+), moderate (++) or strong (+++) [10].

### TUNEL method

The TUNEL (Terminal deoxynucleotidyl-transferase-mediated dUTP Nick End Labeling) method was performed as previously described [35,36]. The sections adhered to silanized slides (3-aminopropyltrithoxysylane - Sigma-Aldrich Chemical Co., St. Louis, USA) were treated with 20 μg/ml proteinase K (Sigma-Aldrich Chemical Co., St. Louis, USA) and immersed in 3% hydrogen peroxide. After immersion in equilibration buffer, the sections were incubated in TdT enzyme at 37°C and, after 1 hour, the reaction was stopped with stop/wash buffer. The sections were incubated in anti-digoxigenin-peroxidase for 30 min and the reaction was revealed with 0.06% 3.3'-diaminobenzidine tetrahydrochloride (DAB - Sigma-Aldrich Chemical Co., St. Louis, USA) and counterstained with Carazi's haematoxylin. Sections of involuting mammary gland, provided by the manufacturer of the Kit, were used as positive controls for the TUNEL method. Negative controls were incubated in a TdT enzyme-free solution.

## Results

### Immunohistochemistry for ERβ

**Figure 1:**
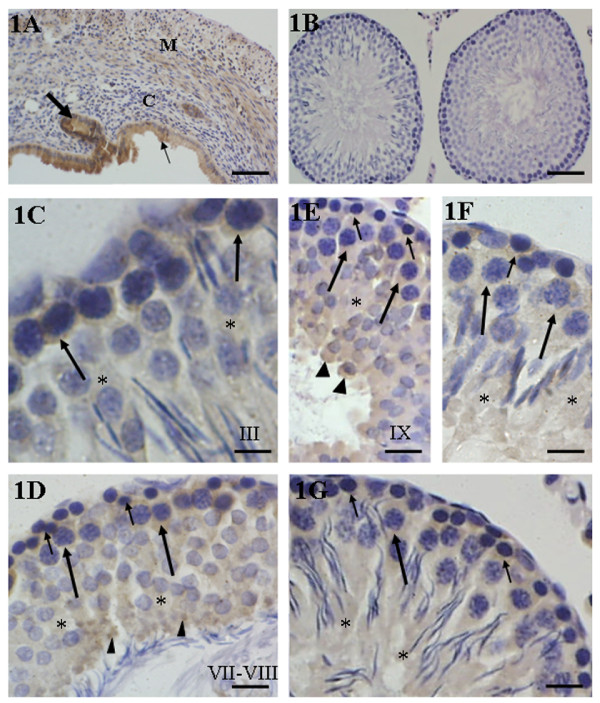
**1A-1G: Immunohistochemistry for detection of ERβ in the uterus (positive control) and in the testes of rats from CG**. In **1A**, strong immunolabeling is observed in the epithelium (thin arrow) and endometrial gland (thick arrow). The connective (C) and muscle (M) tissues show a weak and moderate immunopositivity, respectively. In **1B**, none immunolabeling is observed in the testicular section used as negative control. In **1C-1G**, immunostaining is uniformly observed in the cytoplasm of germ cells of the basal compartment (short arrows), primary spermatocytes (long arrows), round and elongate spermatids (asterisks). An accentuated staining (arrowheads) is observed in the residual bodies (**1D**) and in the caudal portion of early elongating spermatids (**1E**). Scale bars: 100 μm (1A); 60 μm (1B); 10 μm (1C); 21 μm (1D and 1E); 15 μm (1F and 1G).

The uterine sections, used as positive control, revealed an evident immunoreactivity in the epithelium, endometrial glands and muscle tissue (Fig. 1A). In the testicular sections, used as negative control, none immunoreactivity was observed (Fig. 1B).

In CG, an evident cytoplasmic immunostaining for estrogen receptors (ERβ) was observed in the germ cells of the basal compartment (spermatogonia and leptotene spermatocytes) and in zygotene to diplotene spermatocytes in almost all stages (II-XIII). Round spermatids (stages II-VIII) showed a weak cytoplasmic immunostaining (Figs 1C-G). However, a conspicuous staining was noted in the residual bodies derived from elongate spermatids in the tubules at stages VII and VIII and in the caudal portion of elongating spermatids in tubules at stage IX (Figs. 1D and 1E).

**Figure 2:**
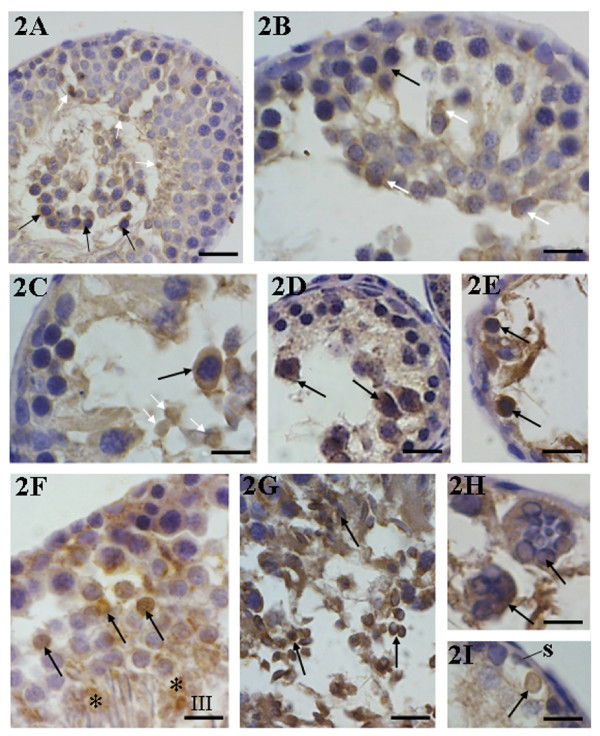
**2A-2I: Immunohistochemistry for detection of ERβ in the testicular sections of rats from CmG**. In **2A**, the seminiferous tubule showing slight alterations exhibits a similar pattern of immunostaining of CG; however, some spermatid nuclei (white arrows) and the detached germ cells filling the lumen (black arrows) are strongly stained. In the damaged tubules (**2B-2E**), a strong immunopositivity (black arrows) is observed in the cytoplasm of primary spermatocytes (**2B-2D**) and germ cells of the basal compartment (**2E**). Note that the immunolabeling is observed in both cytoplasm (**2B**) and nucleus (**2C**) of spermatids (white arrows). In **2F **and **2G**, strong positive immunolabeling is observed in the nucleus of round (**2F**) and elongating (**2G**) spermatids (arrows) and in the residual bodies (asterisks). In **2H**, the immunopositive giant multinucleated cells contain spermatids with peripheral condensed chromatin (arrows). In **2I**, a positive germ cell is inside a vacuole (arrow) next to Sertoli cell nucleus (S). Scale bars: 28 μm (2A and 2D); 20 μm (2B, 2C, 2E-2I).

The seminiferous epithelium of rats from CmG exhibiting lack of elongate spermatids, intraepithelial vacuoles and detached germ cells in the tubular lumen showed a similar immunostaining pattern to those of the control group. However, the nuclei of some round spermatids were also immunolabeled and a strong immunostaining was observed in the detached germ cells filling the tubular lumen (Fig. 2A). In the seminiferous tubules with severe cellular depletion (Table 1), a strong immunopositivity was found in both nuclei and cytoplasm of spermatids, in the cytoplasm of primary spermatocytes and in the germ cells of the basal compartment (Figs. 2B-G). In late spermatids (tubules at stages II-V), a strong positive immunolabeling was observed in the residual bodies (Fig. 2F). Multinucleated giant cells derived from altered round spermatids showed nuclei with peripheral condensed chromatin (typical of apoptosis) and were also strongly positive to the immunoreaction (Fig. 2H). In the maximally damaged tubules, some germ cell nuclei in the basal portion of the epithelium were strongly stained; some of them were inside a vacuole, next to Sertoli cell nucleus (Fig. 2I).

**Table 1:** Scores (weak+, moderate++ and strong+++) of the ERβ immunoreactivity in the germ cells of rats from CG and CmG

Germ Cells	CG	CmG
Spermatogonia	++	+++
Spermatocytes	++	++/+++
Round spermatids	+/++	++/+++
Elongate spermatids	++	+++
Residual bodies	++/+++	+++

### ERβ immunostaining and TUNEL-positive germ cells

**Figure 3:**
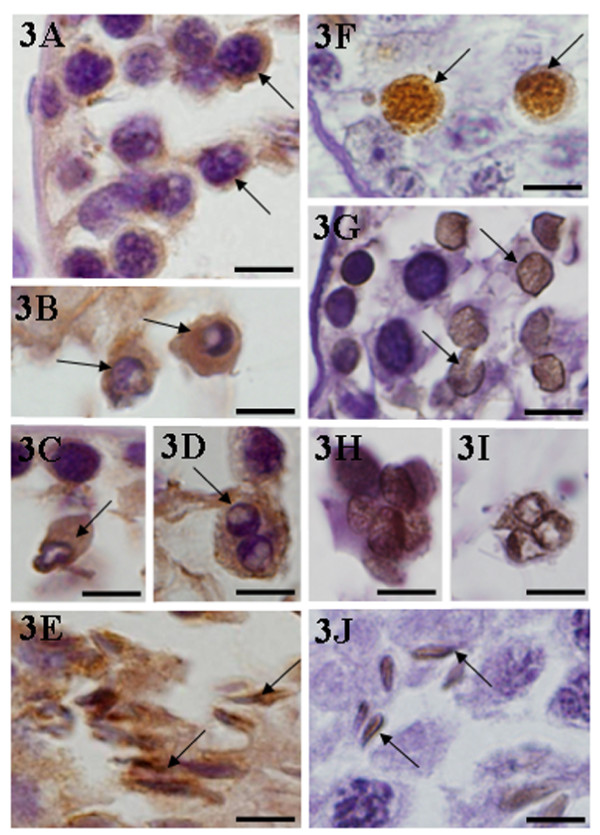
**3A-3J: Immunohistochemistry for detection of ER-β (Figs. 3A-3E) and TUNEL method (Figs. 3F-3J) in the seminiferous tubules affected by cimetidine (CmG)**. In **3A-3E**, spermatocytes (**3A**), round (**3B **and **3C**), elongate (**3E**) spermatids and multinucleated cells derived from spermatids (**3D**) are positive for ER-β immunohistochemistry (arrows). In these cells (**3A-3D**), the nuclei show degenerative aspect, typical of apoptosis. In **3F-3J**, spermatocytes (**3F**), round (**3G**), elongate (**3J**) spermatids and giant multinucleated cells derived from spermatids (**3H and 3I**) are also TUNEL-positive (arrows). Scale bars: 8 μm.

In the damaged seminiferous tubules of cimetidine-treated rats, the TUNEL labeling was observed in the same germ cell types strongly immunostained for ERβ, i.e. primary spermatocytes, round and elongate spermatids and giant multinucleated cells. In the ERβ-immunostained cells, the nuclei also showed degenerative aspects, such as condensed chromatin, typical of apoptosis (Figs. 3A-J).

## Discussion

### ERβ immunoreactivity in untreated rats

In the present study, the cellular ERβ immunolabeling was observed in spermatogonia, primary spermatocytes and spermatids, similarly to previous findings [2,10,18,20,39]. However, the immunoreactivity was always observed in the cytoplasm of these germ cells. Although ER is considered a nuclear receptor, the actual cellular localization of the estrogen receptors has been controversial. Estrogen modulates mitochondrial function, such as ATP production, mitochondrial membrane potential, and calcium concentration [40]. According to Yang [41], ERβ has demonstrated to be a mitochondrial protein rather than a nuclear receptor. Moreover, a number of studies have demonstrated that ERβ is mainly localized extranuclearly [reviewed by [42]]. Additionally to the nucleus, ERβ immunolabeling has been demonstrated in the cytoplasm of different cell types [41-43], including cells of the reproductive system [2,10,19,39]. A diffused immunostaining for ERβ has been demonstrated in the cytoplasm of spermatocytes [2] and in the seminiferous epithelium of bank vole [10]. In the testis of rat ABP transgenic mice, an evident immunostaining has been observed in the cytoplasm of pachytene spermatocytes and metaphase germ cells as well as a less intense cytoplasmic labeling in the elongate spermatids [19]. Moreover, ERβ immunostaining was also detected in human sperm, mainly in the midpiece, at the site where the mitochondria are packaged [16]. Thus, in agreement with these findings, the results of the present study reinforce the cytoplasmic role of estrogen via ERβ in the germ cells.

Studies have demonstrated ERβ immunolabeling in round [2,10,13,18] and elongate spermatids [14]. Moreover, knockout mice that lack a functional aromatase enzyme show a specific defect in the development of spermatids [22]. Therefore, these findings have confirmed that estrogen plays a role in the maturation of spermatids. In the present study, ERβ immunolabeling was observed in the cytoplasm of elongating spermatids, mainly at stage IX. Additionally, the residual bodies derivative from the final steps of spermiogenesis were also immunostained. It has been discussed that the differences concerning positivity and negativity in the cytoplasm of these cells are due to differences in the affinities of the antibodies by the amino acid sequences: N- or C-terminal region [19,39]. In these studies, a less intense but clear labeling was also demonstrated in the luminal compartment, corresponding to caudal portion (cytoplasm) of elongate spermatids and spermatozoa. The present results are compatible with the findings obtained by the authors probably due to the fact that the same amino acids (54-71 amino-terminal region) of rat estrogen receptor-β, such as used by the authors [19,39], were used in the present study.

Additionally to the flagellum, the present findings revealed specific ERβ immunolabeling in the residual bodies of rat spermatids. These results are in agreement to previous studies, in which estrogen receptors immunoexpression have been demonstrated in excess residual cytoplasm [17] of human spermatozoa. In rodents, aromatase (a key enzyme that convert testosterone to estrogen) is observed to move from the Golgi apparatus to the cytoplasm during spermiogenesis and has been immunolocalized in both flagella and residual bodies [reviewed by [4]]. Therefore, the presence of ERβ in the same portions of spermatozoa in which aromatase has been detected reinforces the fact that spermiogenesis, including the formation of residual bodies, are estrogen-dependent processes.

### ERβ immunoreactivity in cimetidine treated rats

In the present study, an accentuated immunoreactivity for ERβ was observed in the damaged tubules by cimetidine. Spermatogonia, primary spermatocytes and spermatids in different steps of spermiogenesis exhibited strong cytoplasmic immunolabeling. In some germ cells, mainly spermatids, a conspicuous nuclear immunoexpression for ERβ was also observed. Cimetidine has demonstrated to cause severe alterations in the seminiferous tubules [31,33-36], including significant decrease in the diameter of tubules at androgen-dependent stages, detachment of spermatids from Sertoli cells [33] and germ cell loss by apoptosis [35]. Thus, a possible interference of cimetidine in the tubular androgenization has been suggested [33,34]. However, cimetidine has also demonstrated to exert a toxic direct effect on the peritubular tissue [31,35]. In a recent study, it has been demonstrated that Sertoli cell-basement membrane interface is structurally affected by cimetidine, leading to Sertoli cells death by apoptosis and significant reduction in the number of these cells [36]. It is known that Sertoli and germ cells are able to convert testosterone into estrogens via aromatase enzyme [1,3,4,8]. Thus, additionally to the antiandrogenic effect of cimetidine and the possible interference on tubular androgenization, the convertion of testosterone to estrogen by aromatase in the damaged tubules by cimetidine may be probably affected, due to Sertoli cell death. Studies have demonstrated a higher estrogen receptor immunoreactivity in different cerebral regions of animals whose source of estrogen was removed by aromatase inhibitors [44,45] or in aromatase knockout (ArKO) mice [46], indicating that estrogen can down-regulates estrogen receptors. Regarding the seminiferous epithelium, a conspicuous ERβ immunolabeling was also detected in the germ cells of rat ABP transgenic mouse; in this rat model, aromatization to estrogen should also be impaired due to significant disruption in the intratubular androgen homeostasis [19]. Thus, it is conceivable to suggest that the increased ERβ immunoreactivity in the germ cells of affected tubules by cimetidine can be related to a possible interference of cimetidine on the tubular androgenization and/or to a possible intratubular aromatase or estrogen deficiency, due to Sertoli and germ cells damage. This is reinforced by the fact that cimetidine induces increase in the FSH levels in men [26] and in rats treated with the same dosage used in the present study [31]. It has been demonstrated that estrogen also plays a role in the feedback control of FSH concentrations [reviewed by [4,47,48]]. In men, the suppressive effect of testosterone on serum FSH is decreased by addition of aromatase inhibitor (testolactone), which in turn inhibits the conversion of testosterone into estrogen [47]. Thus, the increased FSH levels [31] associated to the impairment in the Sertoli cells caused by cimetidine [35,36] support the idea that the ER-beta overexpression in the germ cells of damaged tubules by cimetidine can be related to a possible intratubular estrogen deficiency. However, further studies are necessary to confirm this possibility.

Studies have demonstrated a relationship between germ cell loss by apoptosis and deficiency of estrogen [8] or aromatase [22]. In a previous study, a massive germ cell loss by apoptosis has been demonstrated by TUNEL method in cimetidine-treated rats [35]. The results of the present study also revealed a parallelism between TUNEL-labeling and enhanced ERβ immunoreactivity in the same germ cell types. Thus, while spermatocytes cytoplasm and cytoplasm and nuclei of round and elongate spermatids showed enhanced ERβ immunoreactivity, these same cell types were also positive for the TUNEL method, as previously demonstrated [35] and observed in the present study. Moreover, germ cells strongly ERβ-immunostained were within vacuoles next to Sertoli cells, suggesting phagocytosis, a common event observed during apoptosis [35]. In a recent study made in our laboratory, a similar parallelism between cytoplasmic ERβ overexpression and apoptosis has also been demonstrated in alveolar bone osteoclasts [49]. According to Nilsen et al. [50], ERβ acts as a mediator of apoptosis in cultured neurons. In patients with Alzheimer disease, an increased ERβ immunoreactivity was detected in the cytoplasm of degenerative neuronal cells of hippocampus [51]. Regarding the germ cells, a correlation between ERβ overexpression and apoptosis was also observed either in rat spermatocytes induced to apoptosis by short-term administration of methoxyacetic acid [20] or in rat ABP transgenic mouse [19]. In these studies, the authors suggest that these receptors play a role in the apoptotic process. Thus, the present findings corroborate to the idea that estrogen receptors (ER-beta) are related to the induction of apoptosis in the germ cells.

## Conclusion

The results of the present study point to the cytoplasmic role of estrogen via ERβ in the germ cells. The presence of ERβ immunoreactivity in the flagellum and residual bodies of spermatids reinforces the role of estrogen on rat spermiogenesis.

Cimetidine interferes in the hormonal control of spermatogenesis leading to an accentuated ERβ immunolabeling in the germ cells. Future studies focusing on a possible intratubular aromatase deficiency would be useful to clarify the present findings. The parallelism between ERβ overexpression and apoptosis in the germ cells suggests a possible role of ERβ in the induction of germ cell death.

## Competing interests

The author declares that they have no competing interests.

## Authors' contributions

ESC carried out the treatments of animals, the histological processing, the immunohistochemistry and TUNEL methods and the respective analysis. ESC selected the images and participated, as only author, in the design, writing and final revision of the manuscript.
